# Our New York Letter

**Published:** 1891-10

**Authors:** 


### OUR NEW YORK LETTER.

NEW YORK, Sept. 19, 1891.

Dr. A. Seibert, Professor of Diseases of Children at the New York Polyclinic, has been making clinical experiments with certain drugs in his service at St. Francis Hospital, this city, and as a result of these investigations, has arrived at the following conclusions as to the therapeutic action of these drugs.

In speaking of the diuretic action of *Diuretin*, which is a combination of the obrominnatruim and salicylic soda, he says: The diuretic action of this drug increased from day to day until a period of some two or three weeks had been reached, thus showing that the irritation or stimulation of the kidney epithelium continues for a long time—one of the most desirable virtues that a medicine can possess.

Diuretin is a powerful diuretic in doses of from ten to twelve grammes given during forty-eight hours, in cases of valvular disease of the heart, acute and chronic nephritis. It is also an excellent heart stimulant, increasing blood pressure, reducing abnormal frequency, and correcting irregularities of the heart's action. In a number of cases of fatty heart, cirrhosis of the liver and far advanced cases of parenchymatous nephritis, no action appeared from the use of this drug.

He has been using camphoric acid for the past two years in the treatment of purulent cystitis, pyelitis, and bronchitis, and with very satisfactory results. He has given this drug an extended trial in those forms of bronchial catarrh and broncho-pneumonia complicating and following the severe attacks of influenza. It was administered in eight grain doses from three to six times a day. The first signs of improvement after the use of this drug were noticed invariably on the tongue and in the bronchi. The fetid odor subsided the second day; the tongue became moist, the fur receding, and the small, dry, crepitant rales gave way to vesicular breathing and large moist rales. The area of dulness gradually diminished in size and the appetite returned.

Dr. Seibert does not regard camphoric acid as a specific in bronchitis and the pneumonia of influenza, but he considers it to be a most valuable internal disinfectant of the bronchial mucous membrane.

In the treatment of inflammatory rheumatism, it has been his custom during the past five years to give salicylic soda in injections by the rectum, instead of by the mouth. In acute attacks of rheumatism it is of the utmost importance to give large and frequent doses of salicylic soda, so as to check the progress of the rheumatic germs as speedily as possible, and in this way prevent the development of an endocarditis. In fresh attacks of rheumatism in adults he has given twenty grains of the soda every hour during the first twelve hours, six doses the next day, etc. Aside from the fact of producing nausea, when taken by the mouth, causing distress to the patient and diminishing the power of the already weakened digestion, it is a fact that more of this drug is absorbed and in less time, when given by the rectum than by the mouth. He relates the case of a boy five years of age who had been treated according to this plan. He had been in bed for a period of six months, with swollen knees, ankles, toes and finger joints, being reduced almost to a skeleton with marked anæmia, mitral insufficiency, and such a condition of irritation of the stomach that the very sight of medicine brought on an access of vomiting. This boy was given within a period of about three weeks twenty and one-fourth ounces of the salicylic soda per rectum, and the result was that at the end of that time he could ride a tricycle; the pain left him after the first six doses and his appetite completely returned.

Recently, while workmen were engaged in making excavations in a vacant lot adjoining the so-called "maternity" of an advertising doctor of this city, they suddenly came upon the decomposing bodies of a number of infants, and suspicion was at once directed to the proprietor of the "maternity." The daily papers got wind of the affair, and the usual loud talk was indulged in by the press, but nothing further has been known to come of it. The coroner sat upon the bodies, as every coroner is in duty bound to do, and the affair which was the talk of the town for nine days was very soon forgotten. The "maternity" is still pursuing its career with impunity and the alleged doctor is no doubt glad of the free advertising his iniquitous institution secured. New Yorkers soon learn to forget *trivial* incidents of this nature, for their minds are kept continually occupied with some new sensation.

It may in this connection be of interest to your readers to know something about the *denouement* of the trial of old Dr. McGonigal, the veteran abortionist of this city, whose name was mentioned in a former letter, and the subsequent career of that worthy citizen. His *little* affair was *the* pronounced sensation of the season here and his name was on every one's lips. He was, of course, tried in due form, found guilty of manslaughter, and sentenced to a number of years in State's prison, a practical life sentence for him. This was all right as far as it went and every one approved of the sentence, but here is where the amusing feature of the whole business comes in. McGonigal, strange to say, never spent a single hour behind prison bars, and he is today as free as any man that walks the streets of New York City. His name is also forgotten here and with it the terrible crime of which he was the author.

The regular winter season is now at hand, and students and practitioners are arriving here from all parts of the country, though the new medical law will perceptibly diminish the number of students in attendance of the different undergraduate colleges of this city. The law now insists upon a three years' graded course and a preliminary examination before matriculation. This, of course, will exclude a certain percentage of those who would be otherwise willing to avail themselves of the clinical facilities afforded by the colleges.

The winter session at the New York Polyclinic, which has just commenced, promises to be the most successful one in the annals of this institution. Students are arriving in large numbers from all parts of the country, but notwithstanding the large attendance, the classes are so arranged that every one is enabled to examine and study the abundant clinical material presented.

